# Current Advances in the Development of Diagnostic Tests Based on Aptamers in Parasitology: A Systematic Review

**DOI:** 10.3390/pharmaceutics12111046

**Published:** 2020-10-31

**Authors:** Juan David Ospina-Villa, Alondra Cisneros-Sarabia, Miryan Margot Sánchez-Jiménez, Laurence A. Marchat

**Affiliations:** 1 Instituto Colombiano de Medicina Tropical, Universidad CES, Sabaneta CP 055450, Antioquia, Colombia; jospina@ces.edu.co (J.D.O.-V.); msanchez@ces.edu.co (M.M.S.-J.); 2 ENMH, Instituto Politécnico Nacional, Guillermo Massieu Helguera 239, Fracc. La Escalera, Ticomán, Del. Gustavo A. Madero, Mexico City CP 07320, Mexico; acisneross1903@alumno.ipn.mx

**Keywords:** aptamers, detection test, diagnosis, parasites, SELEX

## Abstract

Aptamers are single-stranded DNA or RNA sequences of 20–80 nucleotides that interact with different targets such as: proteins, ions, viruses, or toxins, through non-covalent interactions and their unique three-dimensional conformation. They are obtained in vitro by the systematic evolution of ligands by exponential enrichment (SELEX). Because of their ability of target recognition with high specificity and affinity, aptamers are usually compared to antibodies. However, they present many advantages that make them promising molecules for the development of new methods for the diagnosis and treatment of human diseases. In medical parasitology, aptamers also represent an attractive alternative for the implementation of new parasite detection methods, easy to apply in endemic regions. The aim of this study was to describe the current advances in the development of diagnostic tests based on aptamers in parasitology. For this, articles were selected following the Preferred Reporting Items for Systematic Reviews and Meta-Analyses (PRISMA) guidelines, with specific inclusion and exclusion criteria. The 26 resulting articles deal with the use of aptamers for the detection of six important protozoa that affect human health. This systematic review clearly demonstrates the specificity, sensitivity and selectivity of aptamers and aptasensors, that certainly will soon become standard methods in medical parasitology.

## 1. Introduction

Aptamers were developed for the first time in 1990 by two different research groups [1,2]. They are single stranded (ss) DNA or RNA molecules of approximately 20–80 nucleotides that recognize and interact with different targets including proteins [3], ions [4], viruses [5], and toxins [6], among others, through no-covalent interactions like van der Waals forces, hydrogen bonds, electrostatic interactions and by complementary forms, due to their unique three-dimensional conformation. Aptamers are usually designed from an in vitro iterative technique named systematic evolution of ligands by exponential enrichment (SELEX) [1,2], through several rounds of selection (seven to ten) that allow one to obtain molecules with high specificity and affinity for the target. Notably, the dissociation constants of aptamers are usually in the nano/picomolar range, which is comparable to those reported for antibodies [7].

Due to their ability of recognizing specific targets, aptamers are often compared to antibodies. However, they present several advantages over them. First, aptamers are generated by chemical synthesis without batch-to-batch variation, ensuring the constant functionality of the molecules. Another advantage is related to the production time, which is relatively short for aptamers (less than three months) compared with the longer production period required for monoclonal antibodies (approximately six months); consequently, the synthesis of aptamers is less expensive, whereas the use of cells and animal models largely increases the cost of antibodies [8]. Moreover, even though aptamers can be denatured, they can return to their original conformation and functionality after the optimal temperature is restored; this characteristic is particularly important since it allows prolonged storage periods and transportation at room temperature, which is not possible with antibodies. Furthermore, aptamers are 10–100 times smaller than antibodies, allowing them to penetrate more easily into the tissues [9]; they can also be modified at their 5′ or 3′ ends, or in their sugar-phosphate chain, to prevent degradation by nucleases or reduce renal filtration, which enhances their stability in vivo and their capability to be used in therapy [10]. Finally, aptamers can be conjugated with biotin, digoxigenin, fluorescent markers, and others—without losing their target affinity and specificity—to be used in diagnostic techniques such as flow cytometry [11], biosensors [12,13,14], enzyme-linked aptamer-based apta-sorbent assay (ELASA) [15], and other multiple applications.

To date, aptamers represent a potential alternative for the diagnosis of cardiovascular diseases [16], cancer [17], and infectious diseases [18,19]. One example is the aptasensor developed by Negahdary and coworkers [16] to detect the cardiac troponin I (TnI) for the early diagnosis of acute myocardial infarction. Results showed a specificity of 81% and a sensitivity of 100% with a detection limit of 10 pg/mL which is better than the conventional enzyme linked immunosorbent assay (ELISA) kit currently employed, indicating that this aptasensor could be useful for the diagnosis of myocardial infarction. Another example is the work of Xi and colleagues [19] that describes the design of an aptamer that recognizes the surface antigen of hepatitis B virus (HBsAg) and can be used in a quimioluminescent aptasensor for hepatitis B diagnostic, in order to replace the conventional ELISA that is a more complex technique and requires a longer processing time; importantly, the aptasensor has a detection limit of 0.05 ng/mL, which is 10-fold lower than the value reported for the ELISA method (0.5 ng/mL).

In the area of medical parasitology, the development of aptamers for diagnosis is in the early steps and parasite infections are still detected by microscopic observation and immunoassays, as well as genome identification by PCR in specific cases. However, some of these methods can give uncertain results or can be difficult to use in the field. In this context, due to their advantages over antibodies, aptamers represent an interesting alternative for the implementation of new parasite detection methods. Thus, the aim of this systematic review was to describe the current advances in the development of diagnostic tests based on aptamers in parasitology.

## 2. Materials and Methods

This systematic review was designed considering the Preferred Reporting Items for Systematic Reviews and Meta-Analyses (PRISMA) guidelines as a reference [20]. An article search was performed in four distinct databases: PubMed, ScienceDirect, Web of Science and Scopus, considering publication dates from January 1990 until August 2020. The searches in all databases were carried out considering the following keywords: “aptamers, diagnosis, diagnostic, parasites, parasitology”, combined with Boolean operators such as “AND” and “OR” and used as follows: “(aptamers AND (diagnostic OR diagnosis OR detection) AND (parasites OR parasitology))”. Article selection was performed considering the presence of two or more of the aforementioned words or parasite names in the title or abstract. Then, the final selection of articles to be included in this systematic review was based on the following inclusion criteria: (1) original research articles using aptamers as diagnostic methods for parasite infections, and (2) texts written in English. While, exclusion criteria were: (1) articles not related to diagnostic methods for parasite infections using aptamers, and (2) review articles or other types of publication. Finally, data of the selected articles were analyzed focusing on two main topics: aptamers characteristics and their utilization for the diagnosis of parasitic infections.

## 3. Results

### 3.1. Article Selection

**Figure 1:**
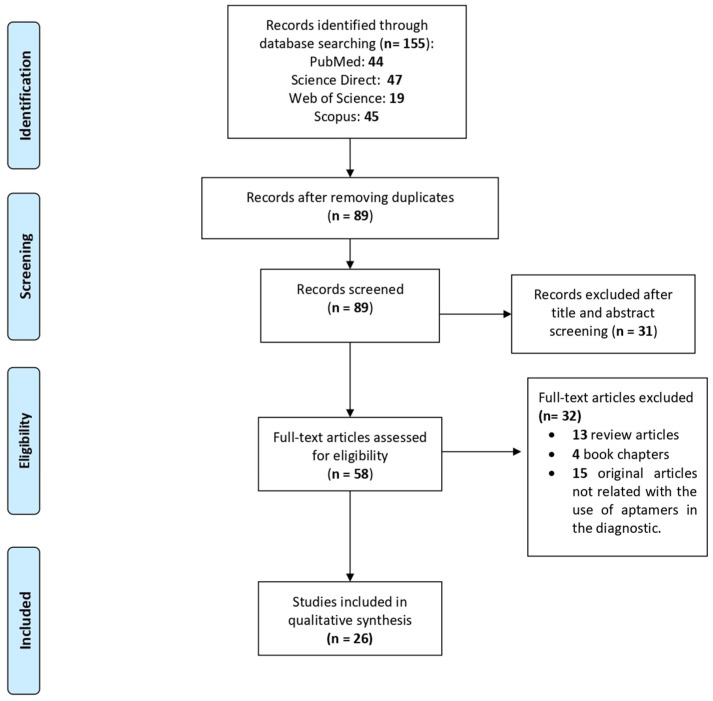
Preferred Reporting Items for Systematic Reviews and Meta-Analyses (PRISMA) flow diagram for article selection included in this systematic review.

A total of 155 articles were obtained from the primary searches, of which 44 were from PubMed, 47 from ScienceDirect, 19 from Web of Science and 45 from Scopus. A group of 66 duplicated papers were removed, leaving a total of 89 articles. Then, we performed a filter step based on the title, abstract and keywords, which reduced the selection to 58 studies. Next, the full text of these 58 articles was reviewed and 32 were excluded because: 13 were review articles, four were book chapters and 15 were not studies related with diagnosis methods for parasite infections. Finally, only 26 articles that met all the inclusion criteria were included in this systematic review (Figure 1). The selected articles are focused on the use of aptamers for the detection of protozoan parasites that affect human health, particularly *Plasmodium*, *Leishmania*, *Trypanosoma*, *Cryptosporidium*, *Toxoplasma*, and *Trichomonas*.

### 3.2. Plasmodium spp.

*Plasmodium* spp. is responsible for malaria, the first leading cause of death from parasites, with 228 million cases and approximately 400,000 deaths each year [21]. Malaria can be cured if detected early, which highlights the need for efficient and easy to use diagnostic methods. Currently, microscopy detection, with or without fluorescent dyes to increase the sensitivity, remains one of the principal methods for malaria diagnosis, although the need for laboratory equipment can be difficult in the field. The use of the polymerase chain reaction (PCR), and colorimetric assays for *Plasmodium* detection is limited by the need of experienced laboratory personnel and considerable sophisticated equipment, while kits based on immobilized labelling antibodies are not authorized for clinical use yet [22]. These observations clearly indicate that better rapid diagnostic tests (RDTs) of malaria are required. Notably, three main groups of investigation have focused their efforts on the development of simple and rapid aptamer-based diagnosis methods for malaria (Table 1).

#### 3.2.1. Aptasensors Using the pL1 Aptamer to Detect the Parasite Lactate Dehydrogenase

A first set of aptasensors for *Plasmodium* detection were designed using ssDNA aptamers raised against the parasite lactate dehydrogenase (LDH) known as an important diagnostic target for malaria. Three of the selected articles used the pL1 DNA aptamer (also called pLDH), obtained by the group of Ban after 10 rounds of selection in a standard SELEX method, based on His-tag *P. vivax* LDH (PvLDH) proteins immobilized on magnetic beads. Interestingly, the pL1 aptamer recognized both PvLDH and *P. falciparum* LDH (PfLDH) with Kd values of 16.8 ± 0.6 nM and 38.7 ± 1.3 nM, respectively [23]. In the first strategy, the same group took advantage of the property of two water-soluble cationic polymers, poly(diallyldimethylammonium chloride) (PDDA) and poly(allylamine hydrochloride) (PAH) to assemble DNA molecules, i.e., pL1 aptamers, into nanostructures, and aggregate gold-nanoparticles (AuNPs) that have unique optical and electronic properties. The method was based on the competitive binding of PDDA or PAH to the pL1 aptamer and AuNPs, depending on the presence or absence of LDH. In the absence of LDH, cationic polymers are sequestered by free pL1 aptamers and thus, cannot aggregate AuNPs. In contrast, the formation of the pL1-LDH complex allows them to accumulate AuNPs, promoting a color change from red to blue that can be easily measured and quantified by using a UV-Vis spectrophotometer and transmission electron microscopy (TEM). After optimization (3.5 nM PDDA or 25 nM PAH, and 10 nM of the pL1 aptamer), the aptasensors were able to detect from 8.7 pM PvLDH for PDDA and 8.3 pM PvLDH for PAH, and 10.3 pM PfLDH for PDDA and 12.5 pM PfLDH for PAH. They were highly selective towards recombinant parasite proteins since they did not detect lysozyme and bovine serum albumin (BSA) that were used as competitive proteins. Importantly, they also permitted the detection of 80 *P. vivax* parasites/μL (92 *P. falciparum* parasites/μL) for PDDA and 74 *P. vivax* parasites/μL (97 *P. falciparum* parasites/μL) for PAH [24]. Then, the same authors showed that the cationic surfactant hexadecyltrimethylammonium bromide (CTAB) is a better candidate for their pL1-based aptasensor, since 4 nM of CTAB with 20 nM of the pL1 aptamer was able to detect from 1.25 pM PvLDH and 2.94 pM PfLDH. This biosensor also identified *Plasmodium* LDH in human serum with limits of detection (LOD) of 10.17 pM and 13.54 pM for PvLDH and PfLDH, respectively, indicating that this simple, sensitive, and selective colorimetric biosensor has a great potential for the diagnosis of malaria [25].

The pL1-aptamer was also used by Geldert and colleagues to develop an interesting fluorescence resonance energy transfer (FRET)-based paper aptasensor. Aptamers were adsorbed on MoS2 nanosheets through the fluorescein amidites (FAM) labeling at their 3′-end. After studying the wettability, and the micro- and nanoscale surface morphologies of five types of paper (two chromatography papers, Whatman Qualitative Filter Paper (No. 1) and Advantec Chromatography Paper (No. 51B), Lens paper (grade 541, VWR, Radnor, PA, USA), A4 printer paper (double A) and coffee filters (Boncafe)), the authors verified that labeled aptamers-MoS2 were distributed throughout the microstructure of the paper and that fluorescence quenching could be quantified from samples dried on paper, as it can be done in classical liquid-based FRET sensors. Finally, strips of paper previously coated with aptamers-MoS2 nanosheets (100 nM pL1 aptamers and 25 μg/mL MoS2 nanosheets) were tested for their reactivity with recombinant *P. falciparum* LDH and unrelated proteins. Surprisingly, printer paper was the only material that showed a significant and specific production of fluorescence on the strip after 30 min of incubation, when aptamers were released from the nanosheet quenchers to bind the parasite LDH (30 nM), making it the most appropriate substrate for this paper-based FRET diagnosis system. This could be due to its lower wettability and smaller pore size, which might maintain the interactions with aptamers-MoS2, allowing for better binding with target molecules. This rational strategy established the basis of a facile, disposable, and low-cost diagnostic FRET-based paper aptasensor that could be easily applied in low-resource areas where infectious diseases such as malaria are endemic [26].

#### 3.2.2. Aptasensors Using the 2008a Aptamer to Detect the Parasite Lactate Dehydrogenase

The group of Tanner developed several bisosensors based on another ssDNA aptamer recognizing the PfLDH, named as 2008s, that was selected after 20 rounds of SELEX using PfLDH immobilized on magnetic beads, and the human homologue for the elimination of nonspecific aptamers. After coupling with AuNPs via a 5′ thiol group, the interaction between PfLDH and 2008s aptamer was determined by isothermal titration calorimetry (ITC), surface plasmon resonance (SPR) and electrophoretic mobility shift assay (EMSA), and showed Kd values of 42 nM, 59 nM and 56 ± 18 nM, respectively. Methods such as TEM evidenced the aggregation of 2008s-AuNPs in the presence of PfLDH. In addition, the loss of the red color was evidenced in the presence of PfLDH in comparison with the observed color in the presence of the human homologue. Interestingly, the LOD of this method was 57 pg/μL PfLDH, therefore this colorimetric assay could be used for the detection of PfLDH in the plasma (2–15 pg/ μL) of patients with malaria [27].

The fact that binding of 2008s to PfLDH does not affect the enzyme activity, prompted the group of Tanner to use 2008s in an aptamer-tethered enzyme capture (APTEC) system able to detect PfLDH in blood samples. For this, lysed blood samples were incubated in 2008s-coated wells to allow PfLDH binding; after washing, LDH activity was easily quantified by the standard colorimetric assay using L-lactate as a substrate and the nitrotetrazolium blue chloride (NTB) dye. The LOD were calculated as 4.9 ng/mL ± 2 ng/mL PfLDH in blood samples, as well as 600 ± 250 parasites/mL for asynchronous parasite culture, and 3500 ± 250 parasites/ mL for ring stage parasites. A comparative assay evidenced that the APTEC test is as efficient as the antibody-based OptiMAL-IT dipsticks to diagnose *P. falciparum* infections in patient blood samples. Moreover, 2008s-coated wells remained able to bind PfLDH in at least six successive APTEC assays, which may allow large population analysis at a lower cost. The authors also demonstrated that it is possible to perform a semi-quantitative APTEC assay by varying the aptamer density to determine PfLDH concentration by visual assessment, which makes it a promising tool to follow parasitemia levels in patients [28]. Additionally, using the same APTEC strategy, Cheung and coworkers in 2018 showed that aptamer 2008s only exhibits a slight signal for *P. vivax* LDH, due to the recognition of a similar cofactor-binding cleft in the enzyme of both *Plasmodium* species, but it did not recognize human LDH and serum albumin, confirming the specificity of 2008s for PfLDH. A remarkable finding of this work is the recognition of the target protein directly in blood samples of infected patients. However, the microtiter plate format limits the general application of this APTEC system in endemic zones, in which the use of electrochemical sensors is generally preferred [29]. Therefore, the same group developed a 3D printed microfluidic biosensor that could be easily used in the field. Briefly, magnetic beads (80 μg) coated with biotinylated 2008s aptamers were incubated with lysed sample of human blood in the incubation chambers, then they were magnet-guided to the wash chamber, and finally, magnetic bead-aptamer-PfLDH complexes were revealed by the formation of an insoluble purple colored formazan dye in the development chamber, that can be captured by a standard mobile-cellular camera phone. This portable biosensor was about 14-fold more sensitive that the original APTEC assay since it allowed for the detection of at least 250 ring stage parasites/μL; moreover, it was specific for *P. falciparum*, which makes it a very promising candidate for a rapid diagnostic test [30].

The 2008s aptamer was also used to develop an original aptasensor based on DNA origami technology. The 5′-end of the 2008s aptamer was ligated to a polyT linker sequence followed by a staple strand that is complementary to 12 different regions of the M13 plasmid sequence. Interestingly, these staple-polyT-aptamers seemed to have a greater affinity for PfLDH that the original 2008s sequence, with higher apparent Kd values from 1090 ± 183 to 647 ± 128 nM in EMSA. Then, modified aptamers were mixed with M13 DNA to form a rectangular DNA origami with at least one aptamer protruding from the structure. Atomic force microscopy imaging revealed that a maximum number of four pfLDH are bound to the DNA origami, and this is probably regulated by the size of the protein and its interaction with two aptamers. Consistent with EMSA data, the LOD was found at 500 nM PfLDH. The DNA origami aptasensor was still able to recognize PfLDH mixed in blood plasma, which is a necessary requirement for diagnosis; moreover, the fact that bound LDH still has enzymatic activity may promote the use of fluorescent molecules to easily visualize the presence of PfLDH in infected samples [31].

Figueroa-Miranda, of the Mayer group, decided to immobilize the 2008s aptamer (0.5 μM) on a gold electrode to develop an electrochemical impedance aptasensor. First, they used 6-mercapto-1-hexanol (6-MCH) as a spacer molecule to block empty spaces, avoid unspecific interactions and allow an accurate interaction between the aptamer and PfLDH. The LOD of the 6-MCH-aptasensor was 0.84 pM PfLDH, which is comparable with other methods previously described for the diagnostic of malaria. The aptasensor also detected the PfLDH in 10-fold diluted human serum with a low LOD of 1.3 pM and a dynamic detection range from 10 pM to 10 nM, indicating that it could be useful for the early stage of *P. falciparum* infection. Moreover, its specificity was established by the use of unrelated proteins, such as bovine and human serum albumin, as well as mouse and human LDH. Interestingly, this 6-MCH-aptasensor can be regenerated with 6 M urea and kept at 4 °C without affecting the binding of the aptamer to the electrode and its affinity for PfLDH, which may reduce costs and facilitates its use in the field [32]. Later, the same group reported improvement of the original design by replacing 6-MCH by polyethylene glycol (PEG). Interestingly, the LOD was reduced to 0.8 pM PfLDH in two-fold diluted human serum, and the detection range was increased (2.3 pM–100 nM). On the other hand, the results of ex situ experiments showed that the PEG-aptasensor incubated with whole human serum presented a LOD of 1.49 pM, with a detection range of 4.5 pM–100 nM, that agrees with the results of in situ experiments. Similarly, as other aptasensors, the PEG-aptasensor only recognized PfLDH but not other proteins present in serum. Considering all the aforementioned information, this aptasensor is an interesting in situ tool that could be used in point-of-care for PfLDH detection [33].

Finally, Kim and Searson used the 2008s aptamer in an aptabiosensor based on aptamer modified magnetic microparticles (MMP) for capture, oligonucleotide-modified quantum dots (QD) for detection, and oligonucleotide-modified AuNPs for signal amplification. First, through a half-sandwich assay using QD conjugated to a cOligos aptamer-cOligo-QD construct (1:6 molar ratio), they proved the recognition of the PfLDH and PvLDH proteins immobilized on a 96-well plate. The next step included the integration of MPP functionalized with aptamers (1:6 ratio) in a MMP-QD sandwich assay which allowed the detection of 0.033–3 ng of PfLDH and PvLDH (0.51 to 46 fmole). Then, for signal amplification and to reduce the LOD, a MMP-AuNP-QD aptamer sandwich assay was designed. Results showed a considerable reduction in the LOD down to 0.66 pg PfLDH and PvLDH proteins, demonstrating a 50-fold higher sensitivity than the MMP-QD aptamer sandwich assay. However, the complete validation of this method still requires the evaluation of clinical samples from patients infected with *Plasmodium* [34].

#### 3.2.3. Aptasensors Using the P38 Aptamer to Detect the Parasite Lactate Dehydrogenase

In 2016, the PfLDH protein was used by the group of Goswami to obtain the P38 ssDNA aptamer after 10 rounds of SELEX selection with the parasite protein immobilized on a polyvinylidene difluoride (PVDF) membrane, as well as human LDH chain A, human LDH chain B and protein-free PVDF membrane for negative selection steps. EMSA study confirmed the binding affinity of P38, showing a Kd value of 0.35 μM. The P38 aptamer was employed for the standardization of an aptasensor based on the use of graphene oxide (GO) as an immobilization matrix. The specificity of the aptasensor for PfLDH protein was confirmed voltammetrically (0.65 V) by measuring the NADH generated from the oxidation of lactate by PfLDH. The P38-aptasensor has other interesting properties, such as an LOD of 0.5 fM with a detection range of 0.5 fM–10 fM; it is able to detect PfLDH in mimicked real samples spiked with 5 fM of recombinant PfLDH, while the sensitivity of most aptasensors generally is in the picomolar range. Furthermore, this aptasensor was able to evidence parasitemia in 20-fold diluted whole samples of patients with *P. vivax* infection, whereas the commercial malaria kits based on antibody based LDH were not. For these reasons the authors propose the use of this aptasensor for samples with a low parasitemia [35].

#### 3.2.4. Aptasensors Using the NG3 Aptamer to Detect the Parasite Glutamate Dehydrogenase

The other parasite biomarker selected for aptamer design is the *P. falciparum* glutamate dehydrogenase (PfGDH) that can be found in patient blood during the sexual and asexual stages of the parasite development. Recently, the group of Goswami obtained the ssDNA NG3 aptamer from 17 rounds of SELEX using the PfGDH immobilized on a PVDF membrane, with additional cycles using a protein-free PVDF membrane and human GDH, respectively, to discard non-specific molecules. In an SPR study, the thiolated NG3 was able to specifically bind to the PfGDH antigen with high affinity (Kd = 79 nM). A monolayer of NG3 aptamer was then chemically immobilized over gold disc electrodes and non-Faradaic electrochemical impedance spectroscopy measurements showed that the aptasensor produced a capacitance response at an optimized frequency of 2 Hz following its binding with the target PfGDH. The LOD was found to be 0.43 pM and 0.77 pM for PfGDH in buffer and in serum, respectively, without significant detection of other malarial biomarkers (PfLDH and PfHRP-II), which makes it a high performance aptasensor for malaria diagnosis [36].The same group also immobilized a self-assembled monolayer of NG3 aptamer on the surface of an inter-digitated gold microelectrode (IDμE) connected to the gate of a field effect transistor (FET), to develop a miniaturized aptamer-based FET biosensor that exhibited a specific and sensitive response to PfGDH in spiked buffer and serum samples, with an LOD of 16.7 pM and 48.6 pM, respectively, which may allow the easy diagnosis of symptomatic and asymptomatic patients with low parasitemia levels in the field [37].

Finally, in a recent study, the same authors used biotinylated P38 and NG3 aptamers to coat magnetic beads (200 μg) to capture *Plasmodium* LDH and PfGDH, respectively, and quantitatively detected them using a cocktail buffer containing lactate and glutamate as substrates, through the conversion of the resazurin dye to resorufin at 600 nm. Data of absorbance and fluorescence intensity revealed that LOD values were 0.55 ± 0.09 pM and 1.72 ± 0.13 pM, respectively, for pan LDH in human blood serum, whereas they were 1.34 ± 0.12 and 1.43 ± 0.14 pM, respectively, in the case of serum spiked with PfGDH. Alternatively, the enzyme reaction was transferred to a Diethylaminoethyl (DEAE)-cellulose surface modified (DSM) chromatographic paper in order to develop an instrument-free portable test based on the use of a standard camera and the ImageJ software for densitometry analysis of the developed color. Results showed that the LOD for pan LDH and PfGDH in the serum sample were 69.25 ± 8.22 pM and 68.75 ± 7.64 pM, respectively. This high sensitivity and the absence of reaction with nonspecific proteins, indicate the potential of this aptasensor for malaria diagnosis [38].

### 3.3. Leishmania spp.

Leishmaniasis, caused by the protozoan parasite of the *Leishmania* genus, is transmitted by the bite of infected sandflies (subfamily phlebotominae). It is estimated that 700,000 to one million new cases occur each year, producing between 26,000 and 65,000 deaths worldwide. There are more than 20 species of *Leishmania* that can cause the three main forms of the disease: visceral leishmaniasis (VL), mucous leishmaniasis (ML), and cutaneous leishmaniasis (CL) [39]. The diagnosis of leishmaniasis diseases involves parasitological, serological and PCR-based methods; however, cost, sensitivity and specificity can be a problem. Here, we described the work of two groups of investigation that developed interesting aptamer-based assays could be interesting alternatives for *Leishmania* diagnosis (Table 1).

#### 3.3.1. Aptamers against Parasite Histone Proteins

*Leishmania* histone H2A has previously been identified as a potential diagnostic marker for VL in canines. Therefore, the group of Gonzalez used the recombinant *L. infantum* H2A protein immobilized on a Ni-NTA resin column to select specific DNA aptamers through the SELEX strategy. After only three rounds of selection, they obtained an aptamer population named SELH2A. Using the enzyme-linked oligonucleotide assay (ELONA) technique to characterize the binding affinity, they confirmed that digoxigenin-labeled SELH2A pool recognizes H2H protein with a Kd = 2.0657 ± 0.652 nM and showed that concentrations higher than 1 pmol of SELH2A are required to detect at least 66 pmol of the recombinant protein, while 4 pmol of the SELH2A population is enough to detect 16.5 pmol of H2A protein. Importantly, SELH2A also slightly recognized the H3 histone that shares structural homology with H2A, but not the related H2B or the ribosomal proteins LIP0, LiP2a and LiP2b. On the other hand, in Slot blot experiments, 1.65 pmol of recombinant H2A transferred onto a nitrocellulose membrane could be detected using 100 nM digoxigenin-labeled SELH2A. Additionally, by means of the Western blot technique, SELH2A had the ability of recognizing the endogenous H2A protein in both nuclear and total extracts of *L. infantum* promastigotes, which indicates that these anti-H2A aptamers might represent a useful tool to develop new diagnostic tests for *Leishmania* infection [40].

The same working group also used the *L. infantum* H3 antigen, another valuable tool for the serodiagnosis and the treatment of VL in canines to isolate a ssDNA aptamer population, named SELH3 after three rounds of SELEX. In an ELONA assay, these SELH3 aptamers (1 μg/mL), were able to detect from 25 ng of H3. Subsequently, the recognition of H3 in nuclear extracts of promastigotes of *L. infantum* was demonstrated by means of a Western blot, using recombinant H3 as a control; the authors argue that some additional bands may correspond to H2A. Additionally, Slot blot results indicated that the SELH3 aptamer population (50 ng/well) was able to significantly detect 15.6 ng (1.4 pmol) of H3 protein in a very significant manner (*p* < 0.001). Altogether, these three different methods confirmed that the population of SELH3 aptamers recognizes H3 with high affinity [41]. After cloning in a T-vector plasmid, two aptamers that could detect recombinant *L. infantum* H3 (rLiH3) with high affinity, were selected and named AptLiH3#4 and AptLiH3#10. Analysis of the 3D structure and degradation by DNAseI in vitro showed that AptLiH3#10 is more stable than AptLiH3#4 since the percentage of degradation was lower for AptLiH3#10 (58.1 ± 19.9%) than for AptLiH3#4 (87.9 ± 7.6%) after one hour of incubation, probably due to the presence of a G-quadruplex motif in AptLiH3#10. Through an ELONA assay, both aptamers were able to significantly detect from 1.25 pmol of rLiH3; moreover, their dissociation constants were very similar (Kd = 0.52 ± 0.05 nM and 0.37 ± 0.05 nM, for AptLiH3#4 and AptLiH3#10, respectively). However, the LOD of AptLiH3#4 and AptLiH3#10 was in the range of 6000 to 9000 parasites, and the authors concluded that some adjustments are still required before these aptamers can be used in a diagnostic test [42].

#### 3.3.2. Aptasensor Using Aptamers against the Parasite Hydrophilic Surface Protein

With the aim of developing a field-portable assay system, Bruno and coworkers performed a cell SELEX and a magnetic beads-based SELEX (10 rounds) to select two distinct aptamers, LmWC-25R raised whole *L. major* promastigotes and LmHSP-7b/11R that recognize the recombinant hydrophilic surface protein (rHSP), respectively. Interestingly, both biotinylated aptamers can specifically reveal the surface of promastigotes by means of confocal microscopy. Therefore, the authors decided to design a sandwich assays using magnetic beads coated with 5′-biotinylated LmWC-25R to capture parasite and the 5′-biotinylated LmHSP-7b/11R as reporter aptamer, and the peroxidase-linked amplex ultra red (AUR) fluorescent assay for development. In this work, the method known as fluorescent assay sensor handheld (FLASH) is presented in a novel way to provide very sensitive and quantitative fluorescence measurements, which can compete with the commercially available Picofluor™ or Quantifluor™ systems (Promega Corp. USA). Indeed, the correlation coefficient between the FLASH method and the Picofluor^TM^ kit was 0.9792 for more than 300 samples analyzed by both systems. This method, which directly detects promastigotes, has wide possibilities for the detection of infected mosquitoes, but it is not useful for human diagnosis since the predominant parasite forms are amastigotes. Once proteins are extracted, the FLASH method can give a result in less than 1 h with an LOD of 100 ng per 2 mL of sample, while real-time PCR can take about 2 h. In addition, FLASH is a portable device that can be taken to the field, where the most vulnerable populations are, without any difficulty [43].

### 3.4. Trypanosoma cruzi

*Trypanosoma cruzi* is an intracellular protozoan parasite and the main etiological agent of Chagas disease that affects around five million people in Central and South America, including Mexico and Colombia. During the acute phase (40–60 days) of Chagas disease, there is a high number of trypomastigotes in the bloodstream of infected individuals, which allows their detection by microscopy and PCR; in contrast, in the early or chronic phase (years), the low number of parasites makes them undetectable by the aforementioned methods [44]. In this context, the group of Debrabant, reported two RNA aptamers that could contribute to the development of new methods for the detection of early or chronic phase of *T. cruzi* infection (Table 1).

In 2012, Nagarkatti and colleagues used the cell-SELEX technique with trypomastigotes of the *T. cruzi* laboratory strain (Tulahuen strain) to obtain RNA aptamers with 2′-F desoxiuridine triphosphate (dUTP) and 2′-F deoxycytidine triphosphate (dCTP) after 12 rounds of selection. All aptamers were able to recognize live *T. cruzi* trypomastigotes with a dissociation constant in the nanomolar range. Among them, Apt68 presented the highest binding affinity (Kd = 7.68 ± 1.63 nM; 20,138 binding sites/parasite). Apt68 is highly specific to *T. cruzi* trypomastigotes, since it did not recognize *T. cruzi* epimastigotes that are in the insect vectors, or trypomastigotes of other related parasites, such as *L. donovani* and *T. brucei*, which is particularly important for diagnostic applications. Furthermore, using the biotinylated Apt68 attached to paramagnetic beads of streptavidin (Apt68-coated beads) in a 96-well flat bottom plate, the authors observed the formation of motile aggregates between Apt68 and live trypomastigotes of *T. cruzi* of Tulahuen strain, and other strains obtained from patient samples, showing that immobilized Apt68 conserved trypomastigote binding activity. The same procedure also allowed for the detection of *T. cruzi* trypomastigotes in human spiked blood samples, even when parasitemia was very low, i.e., 0.33 parasites/mL (five parasites in 15 mL). After recovery by pull-down assay using a magnet, real-time PCR assays were performed to identify *T. cruzi*, observing a Ct value significantly lower in comparison with the negative controls (Ct = 25 ± 0.2 versus Ct = 45, respectively). Altogether, these results demonstrated the potential of Apt68 as a tool to concentrate *T. cruzi* parasites in blood samples with low parasitemia, such as clinical samples collected during the early or chronic phase of infection, to facilitate PCR diagnosis [45].

In another study, the aforementioned workgroup isolated the Apt-L44 ssRNA aptamer after 10 rounds of SELEX using trypomastigote excreted secreted antigens (TESA) of *T. cruzi* coated on a polystyrene 96-well MaxiSorp ELISA plate and preincubation of RNA molecules with uncoated wells and BSA before each round of selection to increase specificity. Additionally, non-specific aptamers were eliminated by incubation with plasma from non-infected mice, before testing selected aptamers with plasma from infected animals. The binding specificity of the biotinylated Apt-L44 was validated by an enzyme linked aptamer assay (ELAA). For this, a polystyrene 96-well plate was coated with TESA or parasite protein extracts, biotinylated Apt-L44 was added at a final concentration of 100 nM, and further detected by the addition of a conjugate streptavidin-alkaline phosphatase and a fluorescent substrate (4-Methylumbelliferyl phosphate). The results confirmed the specific binding of Apt-L44 to TESA and trypomastigote proteins, but not to epimastigote proteins, suggesting that the target of Apt-L44 is only expressed in the mammalian life cycle stage of the parasite. Interestingly, Apt-L44 recognized the TESA of three different strains of *T. cruzi* (Tulahuen, Colombian and 0704), but it did not bind to the TESA of other parasites, such as *L. donovani*. Additionally, ELAA allowed the detection of Apt-L44 target in TESA derived from the plasma of Swiss Webster female mice infected with the Colombian or 0704 strain of *T. cruzi*, or from the plasma of female C57BL/6t mice infected with the Colombian strain during acute (7, 14, 21, 28 days post-infection) and chronic (55, 170, 230 days post-infection) phases of Chagas disease. This study showed the relevance of TESA as biomarkers to demonstrate the presence of live *T. cruzi* trypomastigotes in the host, even when low parasitemia makes them undetectable by other methods. Although some improvements in the ELAA are required to improve TESA detection by Apt-L44, this work proposed an attractive alternative method for an efficient diagnosis of *T. cruzi* infection in patients [46].

### 3.5. Cryptosporidium spp.

*Cryptosporidium* is a protozoan parasite that causes intestinal diseases in animals and humans; notably, this parasite produces gastroenteritis in immunocompetent people and severe disease in immunocompromised individuals. Diagnosis of cryptosporidiosis usually involves examination of patient stool samples with the use of stains or antibodies; immunological methods and antigen detection methods can also be used, as well as PCR [47]. Although *Cryptosporidium* oocysts are the infective form that can be ingested through contaminated unprocessed vegetables or water, there are no standard methods for their efficient detection in food and water to prevent human infection.

Because electrochemical sensors and aptamers can detect biomolecules with a great sensitivity and reproducibility, Iqbal and colleagues aimed to merge and enhance—in a novel way—their capacities to develop an aptamer-based electrochemical biosensor to detect fresh *C. parvum* oocysts in water and/or food. First, they carried out 10 rounds of positive and negative selection in a cell-SELEX protocol to obtain ssDNA aptamers with high specificity for *C. parvum* oocysts, as demonstrated by flow cytometric binding analysis of 5′-FAM labeled aptamers. Then, a set of 14 individual aptamers were selected to be tested in an electrochemical aptasensor. For this, they mixed each anti-aptamer with a thiolated ssDNA primer to construct hybrid molecules that were self-assembled onto a AUNPs-modified screen-printed carbon electrode (GNPs-SPCE). After interaction with oocysts for one hour at 25 °C, an increase in the redox current was detected, and the intensity of the signal was proportional to the concentration of oocysts (0 to 800 oocysts). Interestingly, the LOD was 100 oocysts, which would be sufficient to detect possible *C. parvum* oocysts in fresh samples. It was also possible to demonstrate the specificity and selectivity of the aptasensor using cysts of *Giardia duodenalis* and human serum albumin as negative controls, however, the affinity of aptamers for cysts of other *Cryptosporidium* species needs to be evaluated to be able to propose this aptasensor for controlling food and water quality [48]. A few years later, the same authors published significant improvements in the detection device, reducing the LOD to 50 oocysts. Particularly, through the use of streptavidin-conjugated magnetic beads, the 3′-biotinylated R4-6 aptamer previously identified, was firmly immobilized onto GNP-SPCE, which served as the aptasensor platform [49] (Table 1).

### 3.6. Toxoplasma gondii

*Toxoplasma gondii* is an intracellular parasite with a cosmopolitan distribution that infects most warm-blooded animals. It is transmitted through the consumption of contaminated food and can cause severe ocular, neurological, and systemic symptoms, especially in immunocompromised patients and congenitally infected individuals. Diagnosis involves serologic tests to identify antibodies, direct observation of parasites in samples, as well as PCR detection in specific cases [50].

One of the most important virulence factors identified in this parasite is the rhoptry protein 18 (ROP18), a serine/threonine kinase secreted in the parasitophorous vacuole and the host cell cytosol after invasion that promotes intracellular parasite development. So far, there is no report about the possibility of detecting this protein as a virulence biomarker in human serum. Therefore, Vargas and coworkers performed the SELEX strategy (15 rounds) to select two DNA aptamers, named as AP001 and AP002, with a high ROP18 recognition capacity. Using the direct ELAA, the authors demonstrated that the LOD of both aptamers was 12.5 μg/mL of the total antigens from *Toxoplasma*. With respect to the binding affinity, AP001 showed a higher affinity for recombinant ROP18 (rROP18) with a Kd = 62.7 ± 17.27 nM, whereas AP002 has a Kd = 97.7 ± 22.20 nM. Additionally, AP001 was able to detect rROP18 protein in serum from the minimum concentration of 1.56 μg/mL. In contrast, the LOD of rROP18 was 3.12 μg/mL, in a Sandwich ELAA using anti-ROP18 polyclonal antibodies as a capture agent, indicating that the direct ELAA was a more efficient test for patient analysis. The efficacy of the AP001 aptamer to detect the rROP18 protein in clinical samples was evaluated. Around 60% positivity was found in patients with congenital infection and 22.6% positivity in patients with toxoplasmosis. To date, the discussion remains open for the use of this aptamer as a diagnostic tool and/or predictor of severity in congenital toxoplasmosis [51] (Table 1).

### 3.7. Trichomonas vaginalis

The protozoan parasite *Trichomonas vaginalis* is the causative agent of trichomoniasis, which is the most common non-viral sexually transmitted disease. Trichomoniasis generally produces mild symptoms, but its association with other diseases can increase morbidity rates, indicating the need for efficient detection methods for *T. vaginalis*. Besides parasite culture and microscopic evaluation, several tests based on nucleic acid amplification and immunoassay represent the main options for Trichomoniasis diagnosis [52].

The adhesion proteins on the surface of the parasite play a fundamental role in the consolidation of the infection; among them, the adhesion protein 65 (AP65) is a prominent adhesin located on the parasite cell surface and secreted to the extracellular environment, which could represent a marker of infection detection. Therefore, Espiritu and colleagues used the SELEX protocol to obtain ssDNA aptamers against AP65. One of the most dominant molecules (9%) named AP65_A1, presented a high affinity for AP65 with a Kd = 56 nM by means of SPR. Subsequently, ELA-type assays were performed to determine the ability of the AP65_A1 aptamer to recognize both the AP65 protein and whole *T. vaginalis* parasite; at the same time, an ELISA was performed using the commercial antibody to compare the affinities. Interestingly, AP65_A1 had a higher binding affinity to AP65 than the tested polyclonal antibody, with a Kd value of 1.057 nM versus 12.41 nM, respectively. In addition, the LOD of 8.3 × 10^3^ *T. vaginalis* cells/mL is very close to the minimum amount usually required in commercial antibody-based kits (10^4^ organisms per mL of vaginal fluid), which opens the possibility of using the AP65_A1 aptamer to replace antibody-based kits [53] (Table 1).

## 4. Discussion

**Figure 2:**
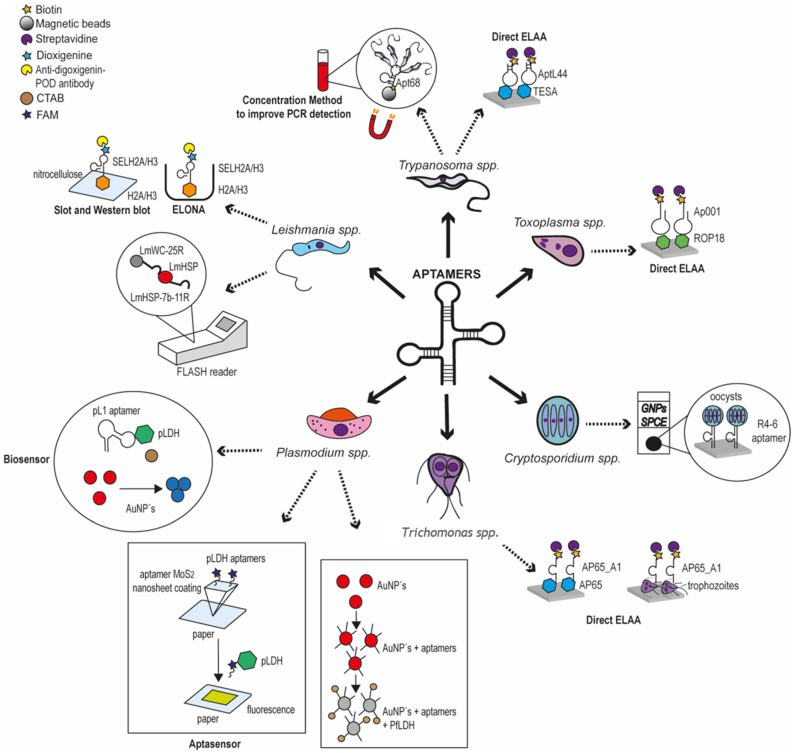
Summary of the main diagnostic tests based on aptamers to identify parasite infections produced by *Plasmodium*, *Leishmania*, *Trypanosoma*, *Cryptosporidium*, *Toxoplasma* and *Trichomonas*.

The remarkable properties of aptamers have promoted technological innovations in many areas of human health, including prevention, treatment and diagnosis. In the medical parasitology field, diagnostic tests based on antibodies are gold-standard methods for the identification of parasite proteins and detection of infection, but the development of alternative systems using more stable molecules, such as DNA and RNA aptamers, may be more suitable for the recognition of pathogens in environmental conditions in which these parasites are endemic. Therefore, it is easy to believe that the classic immunological tools will be replaced by aptamers in the near future. The development of aptamers for diagnostics tests is relatively recent for most parasites that affect human health; indeed, the oldest report selected here was published in 2007 by the group of Gonzalez [40], i.e., only 13 years ago, nevertheless, the results are extremely promising. Our systematic review evidenced that various groups are currently carrying out different aptamer-based approaches to identify distinct parasites. Relatively simple tests, such as ELONA, Slot blot and aptamer-based Western blot assays, among others, as well as more sophisticated aptasensors involving colorimetry, gold particles and spectroscopy, represent very efficient methods for the diagnosis of parasite infections, particularly for the detection of non-immunogenic targets or when antibodies have a low sensibility or a variable efficiency. A summary of the main aptamer-based diagnostic tests developed to identify parasite infections and described in our systematic review is shown in Figure 2.

As expected, the greatest advances in the development of aptasensors in parasitology are related to *Plasmodium* detection, the parasite that is responsible for malaria, the first leading cause of death from parasites; but great progress has also been made for the recognition of *Leishmania*, *Trypanosoma*, *Cryptosporidium*, *Toxoplasma*, and *Trichomonas*. It is worth noting that all the selected articles deal with protozoan parasites, and none are related to metazoan parasites, despite their high prevalence and impact on human health, such as *Schistosoma* spp., the parasitic blood flukes responsible for schistosomiasis, or the mosquito-borne filarial nematodes *Onchocerca volvulus* that cause onchocerciasis (also called “river blindness”) and *Brugia* spp. responsible for lymphedema (elephantiasis), respectively. This may be related to the lower number of groups studying these parasites. It is highly possible that aptamers recognizing proteins of these pathogens will be published soon.

Some of the studies described here involve aptamers that recognize parasite proteins previously known as important diagnostic targets, for example LDH of *Plasmodium* [23,24,25,26,27,28,29,30,31,32,33,34], H2A and H3 histones of *Leishmania* [40,41,42], TESA of *Trypanosoma* [46] and AP65 of *Trichomonas* [53]. Others are based on aptamers raised against the whole parasite itself, such as *Leishmania* promastigotes [43], *Trypanosoma* trypomastigotes [45], and *Cryptosporidium* occysts [48,49]. All these aptamers exhibit high affinity and specificity, which makes them very attractive for parasite detection in patients, particularly in the case of low parasitemia [35,37,45]. In addition, some aptamer-based detection systems may have a role for transmission control through the identification of parasite forms living in the insect vectors [43], while others may be used for water quality control [48,49]. Importantly, the work of Jain and colleagues [35] revealed that an aptasensor can be more performant than an antibody-based kit.

Regarding the signaling method, these aptamer-based diagnostic tests for parasite infections can be categorized into four main groups: (1) optical detection of color change seems to be the simplest one with the advantage that it can be processed through a common smartphone camera making it easy to use in the field [30,38]; (2) the use of a solid phase, for example paper, represents an appealing variation that requires a minimum instrumentation and allows for easy acqusition of rapid results [26,38]; (3) optical detection based on fluorescent molecules exhibits an increased sensitivity with the inconvenience that complex optical equipment is required [43]; and finally, (4) electrochemical detection is an attractive modality due to the potential for miniaturization, portability, and therefore cost reductions [32,33,36,48].

However, these aptamer-based diagnostic tests have some limitations that mainly rely on their application in endemic regions in developing countries. For example, some systems require complex detection techniques, such as spectroscopy or TEM, that may not be available in local laboratories where parasite infections are endemic. Fortunately, investigators also developed efficient and portable devices that use the camera of a standard smartphone for example [30]. Additionally, some of them have only been tested with recombinant proteins and their efficacity for human samples analyses remains to be evaluated [40,41]. Finally, some aptamers are not specific enough and can recognize their target in different species of the same genus, challenging the establishment of a clear diagnosis, such as the pL1 aptamer that recognizes PfLDH and PvLDH [23,24,25]. Further investigations are required to improve the quality of aptamer and aptasensors, but the aptamer technology is here, and it is currently a great opportunity for the development of new diagnostic tools for parasite infections.

Our searches in four databases (PubMed, ScienceDirect, Web of Science and Scopus), using “aptamers, diagnosis, diagnostic, parasites, parasitology” as keywords, retrieved a set of 155 articles, that was further reduced to the 26 articles reviewed here. This quite small number of selected references was expected because of the strict search criteria. Thus, our group recently published a paper about the design of RNA aptamers against an *Entamoeba histolytica* protein [54] that was not selected from the strategy we used for the present review. Moreover, parasite infections are not an area extensive of investigation, like cancer or chemotherapy for example.

Why are there not more published studies on aptamer-based diagnostic tests for parasite infections? Why are there not commercialized aptamer-based diagnostic tests for parasite infections? We can propose several reasons:

- For years, all antibody-based diagnostic methods have focused on the identification of immunogenic proteins. In contrast, a good target for an aptamer does not need to stimulate an immune response. Some of the main criteria for a good target for an aptamer include aqueous solubility, stability, purity, and cost. Therefore, clever approaches are necessary for the identification of new targets with potential for aptamer-based diagnosis.
- The SELEX protocol for the design of aptamers was protected by a patent until 2016 and the window of research opportunity has not been open for very long.
- Although aptamers have numerous advantages over antibodies, the development of commercial products using aptamers takes decades from the identification of a specific aptamer in the laboratory to the development of an aptasensor for parasite identification in patients.

Another remarkable point of this systematic review is that some of the selected articles have been published a decade ago, or more. This might suggest than the detection tests developed in the laboratories were not efficient enough to be applied at large scale in the field. However, we noticed that several of the aptamers and aptasensors described above are currently protected by a patent registration. For example, this is the case for 2008s and pL1 aptamers that recognize *Plasmodium* LDH, and the corresponding diagnostic kits developed by the group of Julian Alexander Tanner and his collaborators [55,56,57]. We also found a patent for the SELH2A aptamer that targets the *Leishmania* H2A histone, that was reported by the group of Víctor Manuel González [58]. Moreover, we discovered that some investigators have moved to the industrial field, establishing relationships with private companies or creating new startups, to continue their work without publishing reports available for the scientific community. For example, the NeXagen company (then renamed as NeXstar) was founded by Larry Gold to develop very important aptamers, such as the one known as Macugen that is the first aptamer-based medicine approved by the US Food and Drug Administration (FDA) and is used for the treatment of age-related macular degeneration (ARMD). Later, two new companies were created: Archemix, in charge of the therapeutic line and SomaLogic, responsible for the diagnostic line. Some other major companies dedicated to the manufacture of aptamers are: NeoVentures founded in 2002 and focused on the commercial use of aptamers in diagnostics, therapies, apheresis and consumer products; Aptagen created in 2004 and located in Jacobus, PA, a suburb of York, that develops aptamers for diagnostics, therapeutics, and bio-industrial applications; Aptus Biotech established in 2010 in collaboration with the Ramon y Cajal Hospital Foundation for the development of aptamers with various biotechnological applications; BasePair founded in 2012 in Houston, Texas, that is focused on healthcare solutions; AptaBharat, the first Aptamer company in India, whose mission is to develop tests for infectious agents detection; AptamerGroup, dedicated to research, diagnostic and therapeutic applications; and AptaTargets, founded in 2014 and located in Madrid, Spain, in collaboration with the Complutense University and the Ramon y Cajal Hospital, that is focused on therapeutic aptamers. As shown on Aptamer society website, (http://aptamersociety.org/aptamer-companies/), more than 40 companies have been created around the world with the aim of developing aptamers and aptamer-based biotechnological applications.

## 5. Conclusions and Perspectives

As far as we know, aptasensors are not commercially available for general clinical use and parasite detection kits still rely on antibodies, beside the limitations of these kind of molecules and the advantages of aptamers. As for all areas of health prevention and care, a large number of studies are required to confirm the feasibility of a new tool; data presented in this systematic review clearly demonstrate the specificity, sensitivity and selectivity of aptamers and aptasensors, and there is no doubt that diagnostic tests based on aptamers will become the standard methods in medical parasitology.

Future perspectives should include improved detection performance and allow for practical application. Thus, it would be interesting to increase the efficiency of biosensors by incorporating chemical modifications in aptamers that enhance the interaction with their target molecule. Avidity could be improved by using combinations of aptamers targeting different molecules, improving the detection limits. Bioinformatics tools such as molecular docking and molecular dynamics could also be very useful to predict and characterize specific interactions between the aptamer and the target, in order to choose the best aptamer candidates to be evaluated in diagnostic tests. In addition, the interdisciplinary work that allows the coupling of aptamers to other technologies, such as electrical or electrochemical sensors has proven to be a great idea to enhance aptamer-based assays. Finally, it is essential to deepen the basic knowledge of parasitic infections and gather all the available information that allows the visibility of parasite biomarkers, in order to select the best target candidates that allow for the most promising aptamer-based methods for the diagnosis of parasitic infections.

Unfortunately, research in parasitology has been declining in recent years and funds are being directed to other human diseases that represent the main health problems worldwide. Government entities and charitable funds that used to be interested in parasitology research have changed their perspectives to support other fields, which certainly contributes to delays in the development of aptasensors for parasite diagnosis. The aptamer technology has to be disseminated so that the scientific community is interested in this promising alternative and begins to use it in their research. It is also necessary to awaken the interest of industries and financing entities for the practical application of this technology in the field of parasitology. The full development of this new technology requires the attention of researchers and public and private companies that contribute to the transition of research to the stage of development of marketable prototypes and, above all, to functional solutions for vulnerable populations in endemic areas.

## Figures and Tables

**Table 1:** Aptamer-based diagnosis methods of infectious diseases caused by parasites.

SELEX			Aptamer			Diagnosis Method	Reference
SELEX Type	Nucleic Acid	Target	Name	Modification	Kd(nM)/Method		
***Plasmodium***							
Standard SELEX	DNA	PvLDH	pL1	ns	16.8 ± 0.6/Fluorescence assay	pLDH aptasensor	[23]
						Colorimetric aptasensors	[24,25]
		PfLDH			38.27 ± 1.3/Fluorescence assay		
						FRET-based paper aptasensor	[26]
Standard SELEX	DNA	PfLDH	2008s	ns	56 ± 18/EMSA 42/ITC 59/SPR	Colorimetric assay	[27]
						APTEC assay	[28]
						APTEC assay	[29]
						3D printed microfluidic biosensor	[30]
				Aptamer ligated to a polyT linker sequence	1090 ± 183 to 647 ± 128/EMSA	DNA origami	[31]
				Aptamer with a 5′-Thiol–(CH_2_)_6_ group	56 ± 18/EMSA 42/ITC 59/SPR	6-MCH-Electrochemical impedance aptasensor	[32]
						PEG-Electrochemical aptasensor	[33]
				ns		Aptabiosensor	[34]
Standard SELEX	DNA	PfLDH	P38	ns	0.35 μM/EMSA	Aptamer-graphene oxide aptasensor	[35]
Standard SELEX	DNA	PfGDH	NG3	Aptamers with a 5′-Thiol–(CH_2_)_6_	79.16 ± 1.58/SPR	Capacitive aptasensor	[36]
						AptaFET biosensor	[37]
Standard SELEX	DNA	PfGDH			79.16 ± 1.58/SPR	Dye Coupled Aptamer-Captured Enzyme Catalyzed Reaction	[38]
		PLDH	P38	ns	0.35 μM/EMSA		
***Leishmania***							
Standard SELEX	DNA	H2A histone	SELH2A	ns	2.065 ± 0.652/ELONA	ELONA, Slot blot, Western blot	[40]
Standard SELEX	DNA	H3 histone	SELH3	ns	0.94 ± 0.19/ELONA		[41]
Standard SELEX	DNA	H3 histone	AptLiH3#4	ns	0.52 ± 0.05/ELONA		[42]
			AptLiH3#10		0.37 ± 0.05/ELONA		
Cell-SELEX	DNA	Live promastigotes	Capture LmWC-25R	ns	ns	FLASH	[43]
Standard SELEX		HSP	Reporter LmHSP-7b/11R.				
***Trypanosoma***							
Cell-SELEX	RNA	Live trypomastigotes	Apt68	Aptamers with fluorinated deoxynucleotides (2′ F-dUTP and 2′ F-dCTP)	7.62 ± 1.63/Binding assay	A concentration method using streptavidin paramagnetic beads	[45]
Standard SELEX	RNA	TESA	Apt-L44		ns	ELAA	[46]
***Cryptosporidium***							
Cell-SELEX	DNA	Oocyst	R4-6	ns	177.5 ± 6.1 μA/Flow cytometric	Aptamer-based electrochemical biosensor	[48]
						DNA- aptasensor coupled with magnetic beads	[49]
***Toxoplasma***							
Standard SELEX	DNA	ROP18	AP001	ns	62.7 ± 17.27/ELAA	Direct or sandwich ELAA	[51]
			AP002		97.7 ± 22.20/ELAA		
***Trichomonas***							
p-SELEX	DNA	AP65	AP65_A1	ns	56/SPR 1.07/ELAA	ELAA	[53]

6-MCH: 6-Mercaptohexanol; APTEC: Aptamer-Tethered Enzyme Capture; ELAA: Enzyme-Linked Aptamer Assay; ELONA: Enzyme-Linked Oligonucleotide Assay; EMSA: Electrophoretic Mobility Shift Assay; FLASH: Fluorescent Assay Sensor Handheld; FRET FET: Field Effect Transistor; HSP: Hydrophilic Surface Protein; ITC: Isothermal Titration Calorimetry; ns: Not Specified; PEG: Polyethylene Glycol; PfLDH: *Plasmodium falciparum* Lactate Dehydrogenase; pLDH: *Plasmodium* Lactate Dehydrogenase; PvLDH: *Plasmodium vivax* Lactate Dehydrogenase; SELEX: Systematic Evolution of Ligands by Exponential Enrichment; SPR: Surface Plasmon Resonance; TESA: Trypomastigote Excreted Secreted Antigens; μA: Microamperes.

## Abbreviations

6-MCH	6-mercapto-1-hexanol
AP65	adhesion protein 65
APTEC	aptamer-tethered enzyme capture
ARMD	age-related macular degeneration
AuNPs	gold nanoparticles
AUR	amplex ultra red
BSA	bovine serum albumin
CL	cutaneous leishmaniasis
CTAB	cationic surfactant hexadecyltrimethylammonium bromide
dCTP	deoxycytidine triphosphate
DEAE	diethylaminoethyl
DSM	DEAE-cellulose surface modified
dUTP	deoxyuridine triphosphate
ELAA	enzyme linked aptamer assay
ELASA	enzyme linked apta-sorbent assay
ELISA	enzyme linked immunosorbent assay
EMSA	electrophoretic mobility shift assay
FAM	fluorescein amidites
FDA	food and drug administration
FET	field effect transistor
FLASH	fluorescent assay sensor handheld
FRET	fluorescence resonance energy transfer
GNPS-SPCE	gold nanoparticles-modified screen-printed carbon electrode
GO	graphene oxide
HBsAg	hepatitis B surface antigen
IDμE	inter-digitated gold microelectrode
ITC	isothermal titration calorimetry
LDH	lactate dehydrogenase
LOD	limits of detection
ML	mucous leishmaniasis
MMP	magnetic microparticles
NTB	nitrotetrazolium blue chloride
PAH	poly Allylamine Hydrochloride
PCR	polymerase chain reaction
PDDA	poly (diallyldimethylammonium chloride)
PEG	polyethylene glycol
PfGDH	*P. falciparum* glutamate dehydrogenase
PfLDH	*P. falciparum* lactate dehydrogenase
PRISMA	preferred reporting items for systematic reviews and meta-analyses
PVDF	polyvinylidene difluoride
PvLDH	*P. vivax* lactate dehydrogenase
QD	quantum dots
RDT	rapid diagnostic test
rHSP	recombinant hydrophilic surface protein
rLiH3	recombinant *L. infantum* H3
rROP18	recombinant rhoptry protein 18
SELEX	systematic evolution of ligands by exponential enrichment
SPR	surface plasmon resonance
ss	single stranded
TEM	transmission electron microscopy
TESA	trypomastigote excreted-secreted antigens
TnI	troponin I
VL	visceral leishmaniasis
